# Sharing and Cooperation of Improved Cross-Entropy Optimization Algorithm in Telemedicine Multimedia Information Processing

**DOI:** 10.1155/2023/7353489

**Published:** 2023-03-06

**Authors:** Hongjiao Wu

**Affiliations:** Henan Vocational College of Tuina, Luoyang 471000, China

## Abstract

In order to improve the efficiency of medical multimedia information sharing, this paper combines cloud computing technology and SOA (service-oriented architecture) technology to build a medical multimedia information sharing system. Building a medical information sharing platform requires integrating information resources stored in information systems of medical institutions and nonmedical information systems related to medical information and forming a huge resource pool. It is important to mine and analyze the information resources in the resource pool to realize the sharing and interaction of medical information. To this end, this paper proposes a gain-adaptive control algorithm with online adjustable parameters and investigates the extension of the mutual entropy optimization algorithm in the control domain and its integrated processing capability in the process of medical multimedia information processing. In addition, this paper constructs a medical multimedia information sharing and collaboration platform with medical multimedia information sharing and telemedicine as the core and verifies the effectiveness of the platform through experiments. The simulation results and comparison results with other systems prove that the system in this paper can realize fast data processing, retrieve and analyze massive data, and meet the demand of remote intelligent diagnosis under the premise of safety and stability. Meanwhile, the system in this paper can help hospitals achieve fast and accurate diagnosis, which has strong theoretical and practical values.

## 1. Introduction

The regional medical multimedia information sharing platform integrates regional medical resources and establishes residents' health files to comprehensively track residents' health status. The whole system provides tracking and management for the health of citizens throughout their lives through high-tech means such as computer technology and modern communication technology and serves every citizen in a timely and accurate manner. With the increasing popularity and deepening of regional medical informatization applications, access control and security audit measures such as firewalls, intrusion detection, intranet monitoring, and antivirus are generally adopted to address security issues such as operating systems, application systems, and network connections. Moreover, the access monitoring and security audit of the database, which is the core of the medical multimedia information system, are equally important [1]. Health information systems audit (ISA) is the process of monitoring, evaluating, and controlling the effectiveness, efficiency, and security of health information systems and their business applications in accordance with recognized standards and guidelines, ensuring that predetermined business objectives are achieved [2]. Specifically, the medical multimedia information system audit takes the medical multimedia information system of an organization such as an enterprise or a government as the audit object. Moreover, through modern auditing theory and management theory, starting from the security of medical multimedia information assets, the integrity of data, and the reliability, effectiveness, and efficiency of the system, it conducts a comprehensive review and evaluation of the entire life cycle process of the medical multimedia information system from development, operation, to maintenance to determine whether it can effectively and reliably achieve the strategic goals of the organization, and to make recommendations for improving and perfecting the organization's control over the medical multimedia information system [3]. The database of the regional medical multimedia information sharing platform stores sensitive medical multimedia information such as patient's disease diagnosis, treatment plan, inspection and test results, and prescription. Illegal access and modification of such medical multimedia information will cause major medical disputes and economic losses. Therefore, it is necessary to use database security audit as a tracking analysis and accountability of security events. By recording and auditing the traces of database operations in detail, the owner of the data can have well-documented access to the database, grasp the usage of the database in time, and adjust and optimize security risks [4].

The database security audit system is mainly used to monitor and record various operational behaviors of the database server. Through the analysis of network data, the system analyzes various operations on the database server in real time and intelligently and records them in the audit database for future query, analysis, and filtering, so as to monitor and audit the user operations of the target database system [5]. It can monitor and audit users' creation, modification, and deletion of database tables, views, sequences, packages, stored procedures, functions, libraries, indexes, synonyms, snapshots, triggers, etc. in the database. The content of the analysis can be accurate to the level of the SQL operation statement [6]. Moreover, it can also intelligently determine the behavior of illegal operation of the database according to the set rules and record and alarm the illegal behavior. Because the database security audit system works on the network where the database host is located in the way of network bypass, it does not need to change any settings of the database system but can track and record the operation of the database and realize the online monitoring of the database [7]. On the premise of not affecting the performance of the database system itself, the online monitoring and protection of the database can detect the illegal operation behavior of the database on the network in time, record, alarm and block in real time and effectively make up for the shortage of the existing application business system in the safe use of the database and provide a strong guarantee for the safe operation of the database system [8].

It is necessary to establish a regional collaborative medical platform, medical multimedia information exchange, and medical multimedia information platform for medical and healthcare, so as to realize the interconnection and resource sharing of medical and health administrative departments and medical and health service institutions in the region. Secondly, it is necessary to promote the optimal combination of regional health resources in the medical multimedia information environment, share health and medical multimedia information resources, and enhance epidemic prevention monitoring, emergency response, and treatment capabilities. In addition, it is necessary to realize a medical multimedia information platform that combines public health (including disease control, supervision, and maternal and child healthcare.) with medical treatment for epidemic monitoring and health supervision and provides district, town, village, family healthcare, and network medical multimedia information services closely related to health [9]. At the same time, it is necessary to promote the medical multimedia informatization of medical services, accelerate the application and promotion of residents' personal health records and electronic medical records, and realize the true life-long preservation and sharing service of personal physiological and medical multimedia information that integrates personal health records and patients' clinical medical multimedia information. Finally, it is necessary to promote medical multimedia information sharing and business collaboration among medical, pharmaceutical, and medical insurance institutions, reduce patient expenses while providing medical quality, and support the reform of the medical system [10].

“Regional Collaborative Medical Platform” is a distributed application across departments, regions, and systems. Data representation and exchange must be standardized and follow a unified standard. The relevant norms and technical standards involved in the project should be sorted out and supplemented as necessary. The basic function of the system includes a unified code maintenance mechanism to adapt to the current situation that the relevant standards are incomplete and the application is ahead of the standard. When necessary, a number of professional standards are formed on the basis of project construction [11].

The application is the purpose, and the security is the guarantee. Therefore, the overall security solution must be certified by an authoritative review, and the security performance of the system must be tested by an authoritative test. In the design and implementation of the “regional collaborative medical platform,” attention should also be paid to making full use of the existing medical informatization achievements, software and hardware resources, and network resources of the regional public health system, especially the HIS system of medical institutions, the public health and emergency medical multimedia information system of each community health service center, and the business medical multimedia information system of specific professional fields. At the same time, it is necessary to integrate resources, gradually unify, combine blocks, and share medical multimedia information [12].

The message system of the regional collaborative medical platform consists of two parts: message server and message client. As a message platform, it enables all applications in the regional collaborative medical platform system to perform corresponding work through the message service platform and to construct and customize different workflows. The message platform is published in the form of server-side and client-side component libraries, and any software system can obtain message services by referencing the methods, properties, and events in this component library [13].

In terms of data security of medical multimedia information, encrypted transmission process and encrypted storage process must be considered. It guarantees that under normal circumstances, applications and users can reasonably view and use medical multimedia information only under the control of medical authority. After illegally obtaining medical multimedia information, the original medical multimedia information data cannot be viewed and obtained normally [14]. The data security design mainly considers the certificate and digital signature verification of the certificate authority CA in the data transmission, the hard disk encryption and compressed storage of medical multimedia information, and the database encryption and compressed storage of medical multimedia information; in the clinical medical authority, a security authorization mechanism is adopted to limit the access of nonmedical personnel and the communication between medical personnel illegal access, etc. [15].

As a part of the telemedicine system, the community home telemedicine monitoring system transmits the collected physiological parameters and video, audio, and image data to the community monitoring center in real time through the communication network to dynamically track the development of the disease to ensure timely diagnosis and treatment. With the popularization of digital TV, telephone, and Internet in the family, telemedicine has rapidly expanded to the family and community, such as remote ECG monitoring, remote midwifery care, remote home care for chronic patients, and telemedicine follow-up. Patients can consult on disease treatment, physical care, diet, etc. in community hospitals [16]. The medical monitoring center terminal can establish contact with family patients through the network, realize the collection and database archiving of regular monitoring data, and can also realize remote observation and interactive diagnosis of patients. The doctor at the medical center can remotely control the rotation of the monitoring equipment at the home end and the zoom of the camera lens through the computer operation, so as to realize the remote observation of the patient. At the same time, they conduct remote diagnosis through voice interaction and can transmit diagnosis results and prescriptions to patients [17].

This paper builds a medical multimedia information sharing system based on cloud computing and SOA technology, which promotes the effective sharing of medical multimedia information resources in the region and enhances the scientific, professional, and refined management of hospital operations.

## 2. The System Design of Regional Medical Data Sharing and Exchange Platform

### 2.1. Regional Medical Cloud Information Sharing Platform System Model Construction

**Figure 1:**
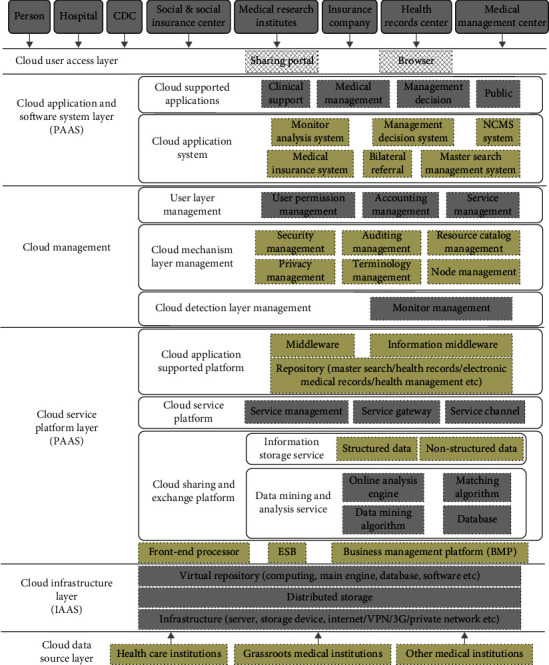
Architecture model of regional medical cloud information sharing platform.

This paper proposes a SOA architecture based on the cloud computing model to build a provincial medical cloud information sharing platform. The platform architecture consists of six layers: cloud data source layer, cloud infrastructure layer, cloud service platform layer, cloud management layer, cloud application and software system layer, and cloud user access layer. Among them, the cloud data source layer provides various healthcare institutions that support the upper layer of cloud computing services. The cloud infrastructure layer provides the various hardware, computing, and storage resources in the underlying layer as services to users. The cloud service platform layer provides the developed and deployed healthcare cloud information sharing platform as a service to the users. The cloud management layer provides functions and technologies for the management and maintenance of all layers of cloud computing services. The cloud application and software system layer provides the various applications and software systems on the platform to users in a web-based manner. The cloud user access layer provides services for on-demand use by platform users through a unified shared portal or browser. The details are shown in Figure 1.

### 2.2. Construction of Computing Center of Medical Cloud Information Sharing Platform

**Figure 2:**
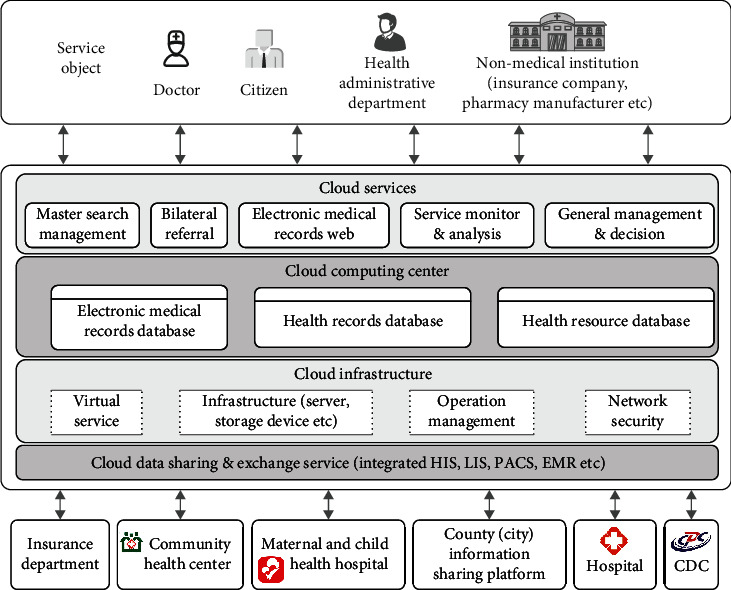
Structure of computing center of medical cloud information sharing platform.

When building a medical information sharing platform, cloud computing can improve the computational analysis ability and data mining ability of massive medical data in the data exchange and sharing platform, find out the association rules between medical data, and carry out in-depth processing and deep-level utilization. It can provide users of the information sharing platform with a large amount of scientific medical data to support healthcare managers in making correct, efficient, and high-quality decisions. The ultimate goal is to improve the quality of medical care and reduce medical costs.  Figure 2 shows the structure of the computing center of the medical cloud information sharing platform based on cloud computing.

### 2.3. Medical Information Sharing and Collaboration Platform Construction

**Figure 3:**
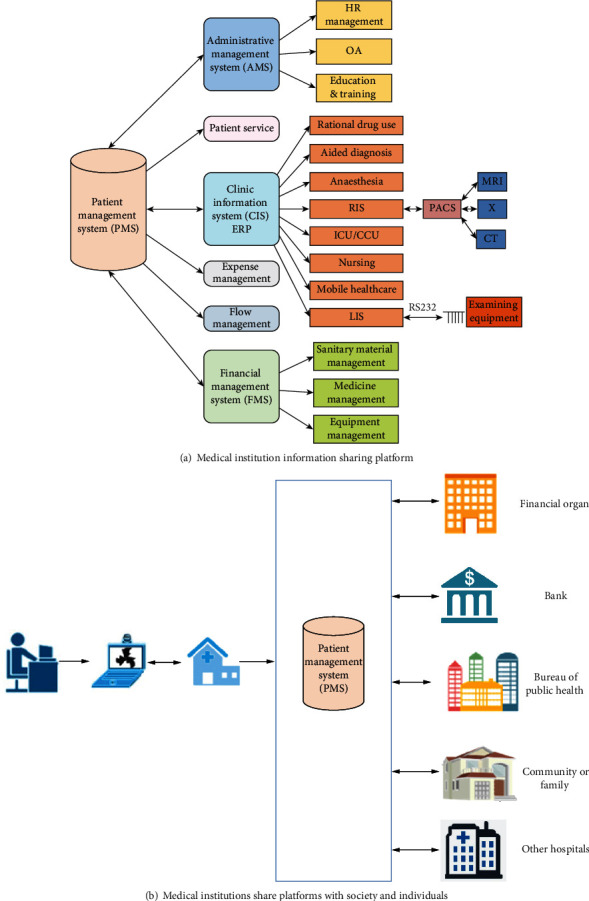
Architecture of medical multimedia information sharing and collaboration platform.

The medical multimedia information sharing and collaboration platform is based on the sharing of medical multimedia information and telemedicine. The shared medical multimedia information includes medical specialties of hospitals, expert information, advanced equipment, cutting edge technology, and health knowledge. The platform improves the quality and efficiency of medical and health services, as well as the analysis, display, and application benefits of medical data, reduces patients' medical costs, and promotes the deepening of medical and health system reform. Medical collaboration is centered on telemedicine and includes medical content such as remote consultation and cross-platform operation. It includes two parts: the information sharing platform of medical institutions and its sharing platform with individuals in society, as shown in Figure 3.

**Figure 4:**
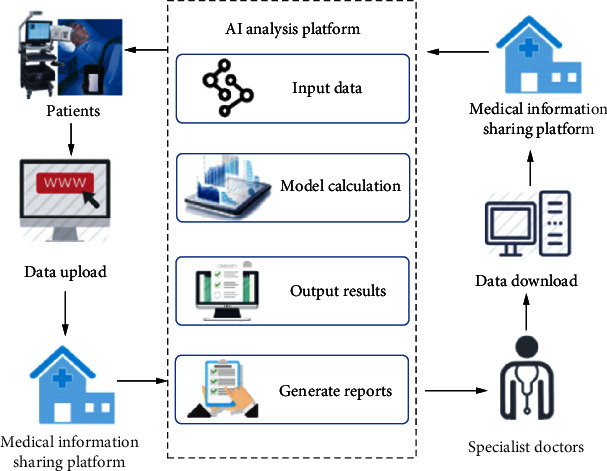
Artificial intelligence-based telemedicine diagnosis.

This paper designs and implements the application of remote diagnosis in medical information sharing and collaboration platform by using information technology tools such as the Internet and artificial intelligence, and deeply combining with clinical diagnosis knowledge and expert experience (see Figure 4). It provides a convenient and feasible path to improve and expand the capability and means of primary medical care services in the field of diagnosis and treatment and promote the sharing of high-quality medical resources across geographical areas.

As in Figure 4, the hospital extracts the data from the film, then transmits the read out data to the human cointelligent analysis platform with the help of network technology, and finally integrates the results of intelligent diagnosis and doctor's diagnosis to make the final diagnosis.

### 2.4. Database Design of Medical Data Sharing and Exchange Platform

**Figure 5:**
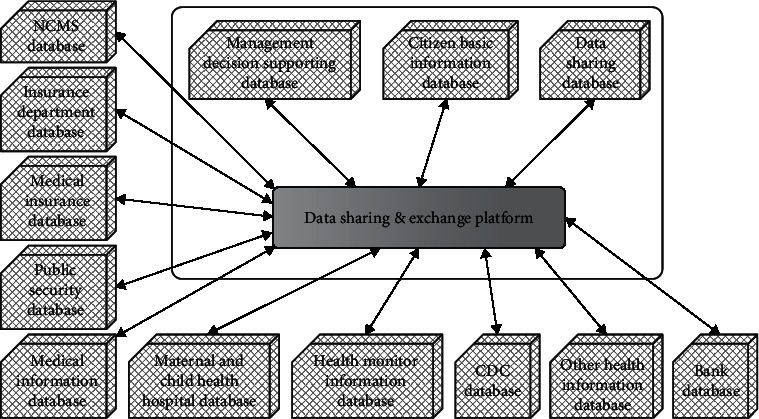
Database distribution diagram of the data center.

The data center consists of three thematic warehouses: the basic health (medical) information database of residents (patients), the database to support management decisions, and the shared database. The data center is connected to medical and health institutions such as community (village) health service institutions, public health institutions, and medical institutions through the medical data sharing and exchange platform and interacts with the information systems of nonmedical and health institutions such as banks, insurance companies, and medical insurance for information resources. It provides data support for the construction of the regional medical information sharing platform and applications such as medical business development, management services, and medical decision making based on the platform and promotes the interconnection of regional medical information resources.  Figure 5 shows the database distribution map of the data center.

### 2.5. Construction of SOA-Based Regional Medical Data Sharing and Exchange Platform

The SOA-based medical data sharing and exchange platform constructed in this paper is based on the unified data exchange standards and enterprise service bus.

#### 2.5.1. Data Exchange Standard

The regional medical data sharing and exchange platform adopts the international common medical data exchange standard HL7 [18] to realize the data representation of each heterogeneous medical information system. Meanwhile, the DICOM standard is used to standardize medical image files [19]. When the medical image files are called and the required services are extracted from them, the standardized medical image files are transferred as SOAP attachments in the form of SOAP messages.

#### 2.5.2. Enterprise Service Bus

ESB (enterprise service bus) [20] plays the role of a platform data center in the regional medical data sharing and exchange platform. All information systems (platforms) that access the platform are connected together through the ESB. The ESB enables a high degree of loose coupling and dynamic interaction between service requestors and providers through business processes and data exchange between each system and the bus.

**Figure 6:**
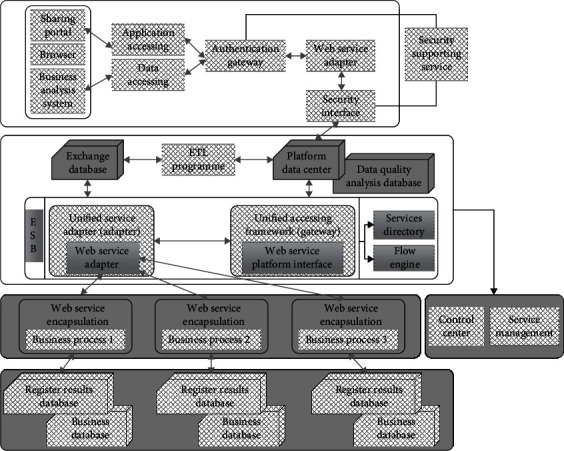
Architecture diagram of regional medical data sharing and exchange platform.

In order to establish a secure, reliable, and scalable information sharing and exchange platform, the framework of the regional medical cloud information sharing platform designed in this paper is shown in Figure 6.

## 3. Medical Multimedia Information Gain Adaptive Algorithm the System of Regional Medical Data Sharing and Exchange Platform

In order to effectively integrate the information resources stored in the information systems of medical institutions and nonmedical institutions related to medical information, this paper proposes a gain adjustment adaptive control algorithm with online adjustable parameters and investigates the extension of the mutual entropy optimization algorithm in the control domain and its integration processing capability in the process of medical multimedia information processing.

### 3.1. Gain Adjustment Adaptive Control Algorithm

**Figure 7:**
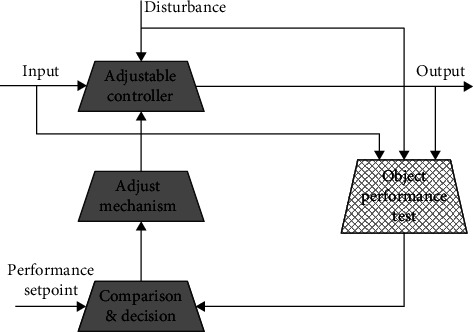
Schematic diagram of the adaptive control system.

The basic idea of various adaptive control methods is consistent. To sum up, the adaptive system includes three necessary components: one is the observation and understanding of the dynamic performance of the process object, the second is the adaptive adjustment mechanism of the controller, and the third is the adjustable controller, as shown in Figure 7.

**Figure 8:**
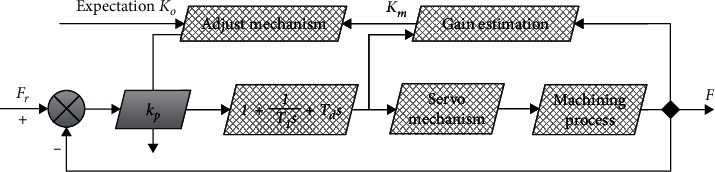
Gain adjustment adaptive control system.

The structure and principle of the gain adjustment adaptive control system are relatively intuitive, and the regulator is designed according to the known variation law of the parameters of the controlled process. When the parameters change due to working conditions and environmental changes, the gain structure of the regulator can be changed according to the prescribed procedure through some variables of the system that can be measured. The basic structure of this controller is shown in Figure 8.

The deviation is

(1) $$\begin{array}{c} e\left(i\right)=F_{r}\left(i\right)-F\left(i\right). \end{array}$$

In the formula, *i* is the *i*th sampling period interval. The command signal *u* from the controller is

(2) $$\begin{array}{c} u\left(i\right)=k_{p}\left(i\right)x\left(1\right)+k_{d}\left(i\right)x\left(2\right)+k_{i}\left(i\right)x\left(3\right). \end{array}$$

Only the control method of online adjustment of proportional gain *k*_p_ is discussed here.

When the depth of cut a changes, the estimator should measure the *K*_s_*alf*^*m*−1^ value in real time. From the model in Section 2, it can be seen that it has a great influence on the open-loop gain. However, estimating this value directly requires the addition of an output path connecting the sensor to the computer and thus requires estimating a value of the process gain *K*_m_, which contains the quantities specified below and is defined as

(3) $$\begin{array}{c} K_{\text{m}}=\frac{60K_{\text{e}}K_{\text{s}}a}{\left(pn\right)f^{m-1}}. \end{array}$$

By definition, at steady state, *K*_m_ is given by

(4) $$\begin{array}{c} K_{\text{m}}=\frac{F}{u}. \end{array}$$

The process gain can be calculated from the *F* and *u* values. Secondly, the controller gain *k*_p_ can be adjusted as follows:

(5) $$\begin{array}{c} k_{p}=\frac{K_{0}}{K_{\text{m}}}. \end{array}$$

In the formula, *K*_o_ is the desired open-loop gain.

### 3.2. Optimal Control Principle Based on Medical Multimedia Information Entropy Measurement

The maximum entropy criterion [21] is called the maximum uncertainty criterion and the maximum entropy method. Any material system always strives for the state of maximum freedom under constraints, leading the system to the most chaotic, complex, and rich state. The maximum entropy criterion means that in a system, if its state is affected by many independent and uniform random factors, the probability distribution of its state should maximize the entropy of this distribution under the constraints that characterize the state of the system. Choosing a distribution less than the maximum entropy means that the difference between the entropy of the distribution and the maximum entropy must come from some additional medical multimedia information. However, this medical multimedia information does not exist objectively and is an unfounded inference, so it is incorrect to choose a distribution less than the maximum entropy. This paper proves it mathematically.

#### 3.2.1. Jaynes' Maximum Entropy [22] Principle

**Theorem 1 .**

In a discrete set *X*, when each event occurs with equal probability, that is, obeys a uniform distribution, its entropy value is the largest, that is,

(6) $$\begin{array}{c} H_{n}\left(p_{1},p_{2},\cdots ,p_{n}\right)\leq H_{n}\left(\frac{1}{n},\frac{1}{n},\cdots ,\frac{1}{n}\right)=\log n. \end{array}$$

Certification: the probability vector is *P* = (*p*_1_, *p*_2_, ⋯, *p*_*n*_), where ∑_*i*=1_^*n*^p_i_ = 1, 0 ≤ *p*_*i*_ ≤ 1.

The random variable is *Y* = 1/*P*, *y*_*i*_ = 1/*p*_*i*_.

According to Jensen's inequality [23], there is

(7) $$\begin{array}{c} E\left(\log Y\right)\leq \log\left(E\left(Y\right)\right),\\ \overset{n}{\underset{i=1}{\sum }}p_{i}\log y_{i}\leq \log\overset{n}{\underset{i=1}{\sum }}p_{i}y_{i},\\ \overset{n}{\underset{i=1}{\sum }}p_{i}\log\frac{1}{p_{i}}\leq \log\overset{n}{\underset{i=1}{\sum }}p_{i}\frac{1}{p_{i}}=\log n. \end{array}$$

That is, when *p*_*t*_ = 1/*n*, the entropy takes the maximum value *H*(*X*) = log*n*.

**Theorem 2 .**

One-dimensional continuous random variable x obeys uniform time distribution in [*a*, *b*], and the source has maximum entropy log(*a* − *b*).

Certification: *p*(*x*) is the probability density of a uniform distribution, *p*(*x*) = 1/(*b* − *a*), and satisfies ∫_*a*_^*b*^*p*(*x*)*dx* = 1, and *q*(*x*) is the probability density function of any distribution and satisfies

(8) $$\begin{array}{c} \int_{a}^{b}q\left(x\right)dx=1.\begin{array}{c} H\left[X,q\left(x\right)\right]-H\left[X,p\left(x\right)\right]\\ =-\int_{a}^{b}q\left(x\right)\log q\left(x\right)dx+\int_{a}^{b}p\left(x\right)\log p\left(x\right)dx\\ =-\int_{a}^{b}q\left(x\right)\log q\left(x\right)dx-\left[\log\left(b-a\right)\int_{a}^{b}p\left(x\right)dx\right]\\ =-\int_{a}^{b}q\left(x\right)\log q\left(x\right)dx-\left[\log\left(b-a\right)\int_{a}^{b}q\left(x\right)dx\right]\\ =-\int_{a}^{b}q\left(x\right)\log q\left(x\right)dx+\int_{a}^{b}q\left(x\right)\log p\left(x\right)dx\\ =\int_{a}^{b}q\left(x\right)\log\frac{p\left(x\right)}{q\left(x\right)}dx. \end{array} \end{array}$$

According to Jensen's principle,

(9) $$\begin{array}{c} \int_{a}^{b}q\left(x\right)\log\frac{p\left(x\right)}{q\left(x\right)}dx\leq \log\int_{a}^{b}q\left(x\right)\frac{p\left(x\right)}{q\left(x\right)}dx=0. \end{array}$$

Therefore, *H*[*X*, *q*(*x*)] ≤ *H*[*X*, *p*(*x*)]. If and only is *p*(*x*) = *q*(*x*), *H*[*X*, *q*(*x*)] = *H*[*X*, *p*(*x*)]. When *p*(*x*) obeys a uniform distribution, the entropy reaches its maximum value.

The core of intelligent control system is to control complexity and uncertainty. When studying a complex control system, it is necessary to establish a simplified model for research or divide the system into several small parts for research and then integrate the partial models into a large model. When simplifying and integrating models, medical multimedia information is inevitably lost. The less medical multimedia information is lost, the closer the model is to reality. Therefore, in the decision-making of uncertainty problems, we choose the scheme that minimizes the loss of medical multimedia information in the system, that is, the smallest uncertainty, that is, the smallest entropy of medical multimedia information. The mathematical model of a stochastic system that changes continuously with time is

(10) $$\begin{array}{c} \frac{dx\left(t\right)}{dt}=f\left[x\left(t\right),u\left(t\right),w\left(t\right)\right]. \end{array}$$

Optimal control is to optimize a certain performance index. The tracking problem is to find a control input *u*(*t*) that interacts with the state variable *x*(*t*) so that the output *c*(*x*, *u*) tracks a given reference signal *r*(*t*) as much as possible. The performance index is

(11) $$\begin{array}{c} S=\int_{0}^{\pi }{\left(c-r\right)}^{2}dt. \end{array}$$

Solving this optimal control problem is to calculate the control variable *u*, which controls the system from the initial state *x*(*t*_0_) to the desired final state *x*(*T*), so that the performance index *S* is minimized. If the output *c* at each moment is the reference signal *r*, the state at each moment is completely determined, the probability of the system being in this state is 1, and the medical multimedia information entropy of this state is zero, that is, *j* = −*P*ln*P* = −1ln1 = 0. If the output *c* of the system at time *t* can vary by *n* units away from the reference signal *r*(*t*), then its uncertainty at that time can be expressed as

(12) $$\begin{array}{c} j\left(t\right)=-\overset{n}{\underset{i=1}{\sum }}p_{i}\ln P_{t}. \end{array}$$

If *c* deviates from *r* from 1 to *n* units with equal probability, namely, *P*_*t*_ = 1/*n*, *i* = 1, 2, ⋯*n*, then

(13) $$\begin{array}{c} j\left(t\right)=-n\cdot \frac{1}{n}\ln\frac{1}{n}=\ln n\left(t\right). \end{array}$$

By integrating the medical multimedia information entropy at each moment from *t*_0_ to *T*, the medical multimedia information entropy can be generated as

(14) $$\begin{array}{c} I=\int_{0}^{T}j\left(t\right)dt=-\int_{0}^{T}\left[\overset{n}{\underset{t=1}{\sum }}p_{1}\ln P_{1}\right]dt. \end{array}$$

The minimum cross-entropy criterion [24] defines a “distance” in the probability space of an incomplete meaning, that is, the interaction entropy, which is used to measure the “gap” between different distributions. This criterion holds that under the constraints of existing medical multimedia information, when the interaction entropy reaches the minimum, the subjective distribution (based on the known medical multimedia information) is the closest to the objective true distribution.

The minimum cross entropy criterion can be described as

(15) $$\begin{array}{c} \min\overset{n}{\underset{t=1}{\sum }}p_{t}\log\frac{p_{i}}{q_{i}},\\ \overset{n}{\underset{i=1}{\sum }}p_{i}=1,\\ \overset{n}{\underset{i=1}{\sum }}p_{i}g_{r}\left(x_{i}\right)=a_{r},r=1,2,\cdots ,m. \end{array}$$

For the minimum interaction entropy criterion, when the prior medical multimedia information does not exist, we take the uniform distribution as the prior medical multimedia information, that is, when there is no prior medical multimedia information, the interaction entropy is

(16) $$\begin{array}{c} D\left(\frac{P}{U}\right)=\overset{n}{\underset{i=1}{\sum }}p_{i}\log\frac{p_{i}}{1/n}=\log n-\left(-\overset{n}{\underset{i=1}{\sum }}p_{i}\log p_{i}\right)=\log n-H\left(P\right). \end{array}$$

In this way, when the interaction is the smallest, the entropy actually reaches the maximum value.

### 3.3. Optimal Control of Medical Multimedia Information Processing Process Based on Mutual Entropy

For the entire medical object, the specific goal of control is to ensure that the output value of the control object is the closest to the given ideal output value. According to medical multimedia information theory, for system *A* and system *B*, the degree of difference between their states *A*_*i*_ and *B*_*i*_(*i* = 1, ⋯, *n*) can be measured by the Kullback–Leibler distance [25] definition.

(17) $$\begin{array}{c} D=\overset{n}{\underset{i=1}{\sum }}\left\{A_{i}\ln\frac{A_{i}}{B_{i}}+\left(1-A_{i}\right)\ln\frac{1-A_{i}}{1-B_{i}}\right\}. \end{array}$$

Specific to the medical multimedia information processing process, when the difference between the expected cutting force value and the actual cutting force value is given, the closeness between the output value of the control system and the ideal output value can be expressed as mutual entropy.

(18) $$\begin{array}{c} \text{ entropy}=\overset{N}{\underset{i=1}{\sum }}\left\{F\left(t\right)\ln\frac{F\left(t\right)}{F_{r}\left(t\right)}+\left(1-F\left(t\right)\right)\ln\frac{1-F\left(t\right)}{1-F_{r}\left(t\right)}\right\}. \end{array}$$

In this formula, the operator log is used to replace the operator ln. The essence of the two is the same, but the unit is different. If *e* is the base, the unit of entropy obtained is knight (Nat), and ln2 = 1Nat is specified. If the base is 10, the entropy is obtained by the operator log, and its unit is Det, and lg2 = 1Det is specified. Obviously, there are conversion formulas between units: 1bit = 0.693Nat = 0.301Det.

The partial procedure of parameter self-adjustment based on mutual entropy is shown below. Among them, e^−4^ is the set control precision. First, the system output value is divided by a number slightly larger than the expected output value, trying to keep it in the range (0, 1).

## 4. Experiments and Result Analysis

### 4.1. System Performance Experiment

In order to reduce the impact of instability on the system during network transmission, the experiments took the time measurement of block height (the distance between the block and the Genesis block) to test the system performance. Using such a metric, the timestamp of a transaction confirmation is the block height it was mined to. Also, using the block height metric can control the time cost variables to two, transaction congestion and transaction fees. Even though the time cost is still somewhat unpredictable due to delays, it is easier to find out the reasons for long waits and delays through comparative analysis.

**Figure 9:**
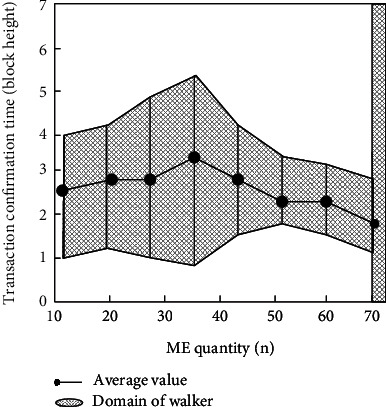
Relationship between policy deployment confirmation time (block height) and the number of MEs.

This paper measures the confirmation time of invoking a smart policy, increasing the number of MEs (multiple elements), as an approximate method for estimating policy complexity. Each measurement has been repeated 10 times. Likewise, this paper gives a smart policy that invokes a single smart AM (architecture model) constructed from the combination of simple single-attribute MEs that are always true on the smart AM's return value. Furthermore, to avoid introducing artificial congestion in the experiments, each policy deployment is set at a time interval of one block.  Figure 9 shows the time results of deploying the strategy in terms of block height.

**Figure 10:**
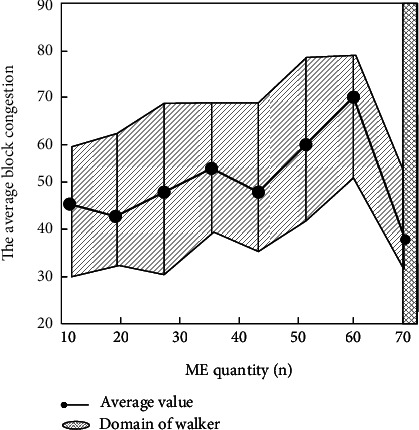
Relationship between average block congestion and number of MEs.

Observing Figure 9, it can be seen that the average time measured experimentally is shorter (always less than 4 blocks) and has no obvious relationship with the number of MEs. To further demonstrate this point, the average block congestion in Figure 9 is measured in the following experiments. To measure the average block congestion of transactions, the value was measured 10 times for different numbers of MEs. The results are shown in Figure 10.

**Figure 11:**
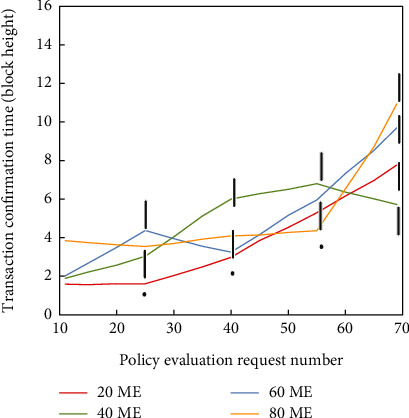
The effect of different ME numbers on the relationship between the average confirmation time of policy evaluation and the number of evaluation requests.

This paper measures the execution time of increased requests and MEs in smart policies. For each fixed number of MEs, this paper performs five experiments under different evaluation requests, and the number of evaluation requests is 20, 40, 60, and 80, respectively.  Figure 11 shows the average execution time of policy evaluation represented by the block height difference, and it can be seen that the average time spent generally increases with the number of evaluation requests. It is theoretically possible that in each block there will be more evaluation transactions competing for the same block limited space, resulting in increased time.

The above experiments prove that the control algorithm in this paper can make the system obtain better dynamic performance in a shorter time, and the transition process has a shorter time and stronger robustness.

### 4.2. Simulation Experiment

**Figure 12:**
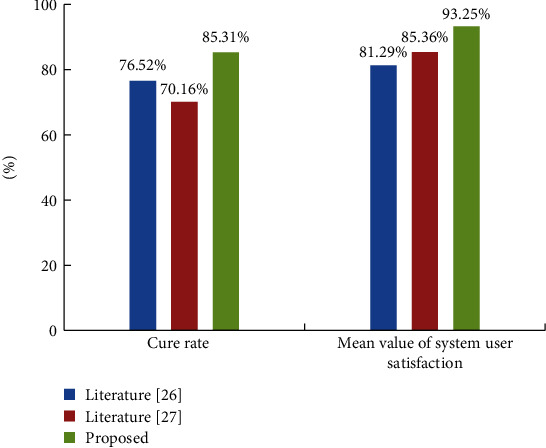
Comparison results of disease cure rate and the mean value of system user satisfaction with three systems.

After the platform was deployed online, it was successfully interfaced with several hospitals to receive image information.  Figure 12 shows the system in literature [26] and the system in literature [27] as a comparison, which was applied to the study area for simulation experiment. All other conditions being equal, the disease cure rate and the mean value of system user satisfaction were compared among the three systems.

The analysis of the data in Figure 12 shows that the system in this paper increases the cure rate and user satisfaction and reduces the cost to some extent. This is mainly due to the fact that the AI-based medical information sharing platform of this paper can achieve rapid diagnosis by using expert remote diagnosis, which reduces the need for patients to seek medical treatment in different places and enhances the economic benefits of medical institutions.

In this experiment, 5 groups of experiments were set up, and the numbers of medical institutions were 5, 10, 15, 20, and 25, respectively. The information sharing communication connection and security operation conditions of these five groups with different numbers of offsite medical service providers were tested separately, and the results are shown in Table 1.

**Figure 13:**
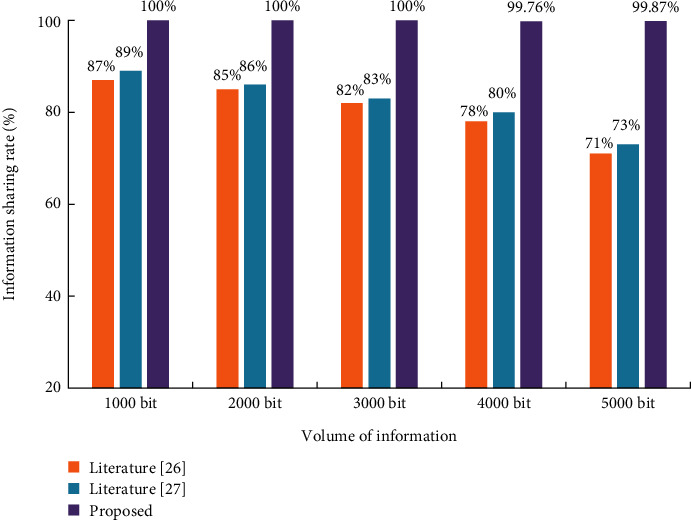
Comparison results of information sharing rate with three systems.

The experiment results in Table 1 show that the information sharing system designed in this paper has advantages in information sharing stability and security compared with the other two medical information sharing systems. According to the experiment method, the information sharing rate under different medical information conditions was tested. Five groups of experiments were set up, and the results are shown in Figure 13.

The experiment results in Figure 13 show that the information sharing rate of this paper's platform does not change too much as the amount of medical information increases. When the volume of information exceeds 4000 bit, the information sharing rate of this paper's platform shows a small decrease but stays above 99%, while the other two platforms only have 80% or less.

## 5. Conclusion

This paper combines cloud computing technology and SOA technology to build a medical multimedia information sharing system to promote the effective sharing of regional medical multimedia information resources. The purpose of establishing a regional collaborative medical platform and medical multimedia information exchange and medical multimedia information platform is to build a patient-centered digital hospital with clinical applications and decision support as the main line by using advanced technologies such as medical multimedia information network. At the same time, network technology, big data, and artificial intelligence are applied to improve the transmission, processing, and storage of medical multimedia information between large- and medium-sized hospitals and community health services and to organically integrate community medical diagnosis and treatment with healthcare services. In addition, the system in this paper can realize telemedicine diagnosis, which can not only assist in primary diagnosis and treatment but also effectively promote the sinking of medical resources, which is of great significance to promote graded diagnosis and treatment. According to the simulation results, it can be seen that the medical multimedia information sharing system proposed in this paper has good effects.

## Figures and Tables

**Table 1:** Information sharing communication connection stability and information security status of different number of offsite hospitals.

Experiment metrics	System type	Number of offsite hospitals				
		5	10	15	20	25
Number of hospitals with stable information sharing	Literature [26]	3	8	10	14	15
	Literature [27]	4	7	11	16	20
	Proposed	5	10	15	20	25

Number of hospitals with secure information sharing	Literature [26]	4	6	12	13	16
	Literature [27]	2	8	11	15	14
	Proposed	5	10	15	20	25

## Data Availability

The labeled data set used to support the findings of this study is available from the corresponding author upon request.

## References

[1] Li Y., Zhang Z., Xia S., Chen H. H. (2021). A load-balanced re-embedding scheme for wireless network virtualization. *IEEE Transactions on Vehicular Technology*.

[2] Rawat D. B. (2019). Fusion of software defined networking, edge computing, and blockchain technology for wireless network virtualization. *IEEE Communications Magazine*.

[3] Han Y., Tao X., Zhang X., Jia S. (2020). Hierarchical resource allocation in multi-service wireless networks with wireless network virtualization. *IEEE Transactions on Vehicular Technology*.

[4] Raveendran N., Gu Y., Jiang C. (2021). Cyclic three-sided matching game inspired wireless network virtualization. *IEEE Transactions on Mobile Computing*.

[5] Rawat D. B., Alshaikhi A., Alshammari A., Bajracharya C., Song M. (2019). Payoff optimization through wireless network virtualization for IoT applications: a three layer game approach. *IEEE Internet of Things Journal*.

[6] Li M., Yu F. R., Si P., Zhang Y. (2018). Green machine-to-machine communications with mobile edge computing and wireless network virtualization. *IEEE Communications Magazine*.

[7] Jiang H., Wang T., Wang S. (2018). Multi-scale hierarchical resource management for wireless network virtualization. *IEEE Transactions on Cognitive Communications and Networking*.

[8] Ho T. M., Tran N. H., Le L. B., Han Z., Kazmi S. A., Hong C. S. (2018). Network virtualization with energy efficiency optimization for wireless heterogeneous networks. *IEEE Transactions on Mobile Computing*.

[9] Cao H., Zhu H., Yang L. (2020). Collaborative attributes and resources for single-stage virtual network mapping in network virtualization. *Journal of Communications and Networks*.

[10] Javadpour A. (2019). Improving resources management in network virtualization by utilizing a software-based network. *Wireless Personal Communications*.

[11] Wei J., Yang K., Zhang G., Lu X. (2019). A QoS-aware joint power and subchannel allocation algorithm for mobile network virtualization. *Wireless Personal Communications*.

[12] Javadpour A., Wang G. (2022). cTMvSDN: improving resource management using combination of Markov-process and TDMA in software-defined networking. *The Journal of Supercomputing*.

[13] Sapavath N. N., Rawat D. B. (2020). Wireless virtualization architecture: wireless networking for Internet of Things. *IEEE Internet of Things Journal*.

[14] Zhou Y., Yu F. R., Chen J., Kuo Y. (2019). Robust energy-efficient resource allocation for IoT-powered cyber-physical-social smart systems with virtualization. *IEEE Internet of Things Journal*.

[15] Lu X., Ni Q., Zhao D., Cheng W., Zhang H. (2019). Resource virtualization for customized delay-bounded QoS provisioning in uplink VMIMO-SC-FDMA systems. *IEEE Transactions on Communications*.

[16] Miao Z., Wang Y., Han Z. (2019). A supplier-firm-buyer framework for computation and content resource assignment in wireless virtual networks. *IEEE Transactions on Wireless Communications*.

[17] Xu S., Li P., Qi F. (2019). Load-balancing and QoS based dynamic resource allocation method for smart gird fiber-wireless networks. *Chinese Journal of Electronics*.

[18] Jones J., Gottlieb D., Mandel J. C. (2021). A landscape survey of planned SMART/HL7 bulk FHIR data access API implementations and tools. *Journal of the American Medical Informatics Association*.

[19] Aiello M., Esposito G., Pagliari G., Borrelli P., Brancato V., Salvatore M. (2021). How does DICOM support big data management? Investigating its use in medical imaging community. *Insights Into Imaging*.

[20] Hu S. (2022). Research on medical multi-source data fusion based on big data. *Recent Advances in Computer Science and Communications (Formerly: Recent Patents on Computer Science)*.

[21] Cabrera J. S., Lee H. S. (2020). Flood risk assessment for Davao Oriental in the Philippines using geographic information system-based multi-criteria analysis and the maximum entropy model. *Journal of Flood Risk Management*.

[22] Baggenstoss P. M. (2018). Beyond moments: extending the maximum entropy principle to feature distribution constraints. *Entropy*.

[23] Adil Khan M., Hanif M., Abdul Hameed Khan Z., Ahmad K., Chu Y. M. (2019). Association of Jensen’s inequality for s-convex function with Csiszár divergence. *Journal of Inequalities and Applications*.

[24] Geyer S., Papaioannou I., Straub D. (2019). Cross entropy-based importance sampling using Gaussian densities revisited. *Structural Safety*.

[25] Zhou T., Peng D., Xu C., Zhang W., Shen J. (2018). Adaptive particle filter based on Kullback–Leibler distance for underwater terrain aided navigation with multi‐beam sonar. *IET Radar, Sonar & Navigation*.

[26] Zhai Y., Gao J., Chen B. (2020). Design and application of a telemedicine system jointly driven by videoconferencing and data exchange: practical experience from Henan Province, China. *Telemedicine and e-Health*.

[27] Chand R. D., Kumar A., Kumar A., Tiwari P., Rajnish R., Mishra S. K. Advanced communication technologies for collaborative learning in telemedicine and tele-care.

